# Polyclonal evolution of lymphoproliferative disorders in XLP1

**DOI:** 10.70962/jhi.20250202

**Published:** 2026-03-12

**Authors:** Dan Tomomasa, Akira Nishimura, Kenichi Yoshida, Yui Namikawa, Doo Ri Kim, Naoki Sakata, Kenichi Sakamoto, Takashi Taga, Yuta Sakai, Yasuhiro Ikawa, Toshiaki Ishida, Areum Shin, Keon Hee Yoo, Yae-Jean Kim, Seishi Ogawa, Akihiro Hoshino, Tomohiro Morio, Masatoshi Takagi, Hirokazu Kanegane

**Affiliations:** 1 Department of Pediatrics and Developmental Biology, Institute of Science Tokyo, Tokyo, Japan; 2 Division of Cancer Evolution, National Cancer Center Research Institute, Tokyo, Japan; 3 Department of Pediatrics, Samsung Medical Center, Sungkyunkwan University School of Medicine, Seoul, Korea; 4 Department of Pediatrics, Kindai University Faculty of Medicine, Osaka-Sayama, Japan; 5 Department of Pediatrics, Shinshu University School of Medicine, Matsumoto, Japan; 6 Department of Pediatrics, Shiga University of Medical Science, Shiga, Japan; 7 Department of Pediatrics School of Medicine, Institute of Medical, Pharmaceutical and Health Sciences, Kanazawa University, Kanazawa, Japan; 8 Department of Pediatrics, Kanazawa Medical University, Ishikawa, Japan; 9 Department of Hematology and Oncology, Kobe Children’s Hospital, Kobe, Japan; 10 Department of Pathology and Tumor Biology, Graduate School of Medicine, Kyoto University, Kyoto, Japan; 11 Institute for the Advanced Study of Human Biology, Kyoto University, Kyoto, Japan; 12 Kindai University Faculty of Medicine, Osaka, Japan; 13 Department of Child Health and Development, Institute of Science Tokyo, Tokyo, Japan

## Abstract

X-linked lymphoproliferative syndrome type 1 (XLP1) is an inborn error of immunity caused by pathogenic variants in *SH2D1A* and is frequently complicated by Epstein-Barr virus (EBV)–associated lymphoproliferative disorders (LPDs). However, cases of LPD without EBV infection have been reported and remain poorly understood. We investigated tumorigenesis mechanisms through transcriptomic profiling and somatic variant analysis in tumor samples from six patients with XLP1. Pathogenic variants were identified in two: one developed two distinct LPDs harboring *CARD11*/*GNA13* and *MECOM* variants, while the other carried *IRF4*, *P2RY8*, *KRAS*, and *CCND3* variants. Transcriptome analysis of three tumors, compared with diffuse large B cell lymphoma from patients without an underlying immune defect, revealed a distinct expression profile. Gene Ontology analysis showed upregulation of adaptive immune response genes, including various *IgH* and *TCR* genes, suggesting polyclonal lymphocyte proliferation. Overall, LPD associated with XLP1 may originate from polyclonal lymphocyte expansion, either in the presence or absence of EBV infection, and subsequently progress to malignancy through somatic variants.

## Teaser

We investigated tumorigenesis mechanisms through transcriptomic profiling and somatic variant analysis in tumor samples from six patients with XLP1. LPD associated with XLP1 may originate from polyclonal lymphocyte expansion, either in the presence or absence of EBV infection, and subsequently progress to malignancy through somatic variants.

## Graphical Abstract

**Figure:**
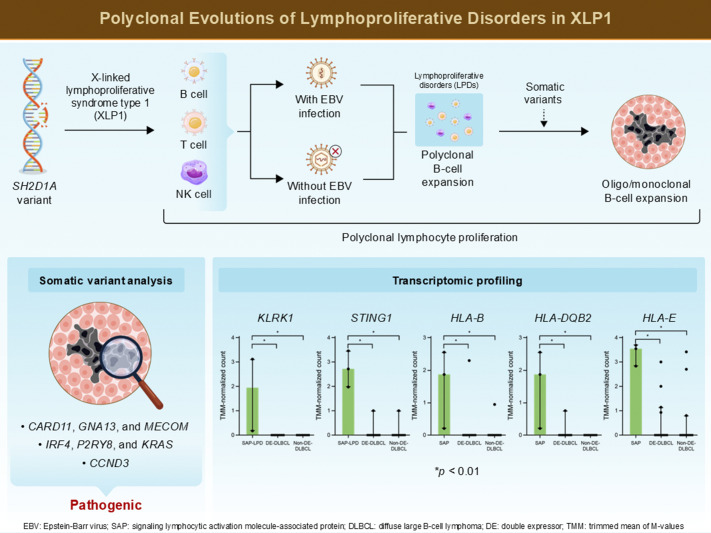

## Introduction

Signaling lymphocytic activation molecule-associated protein (SAP) deficiency, also known as X-linked lymphoproliferative syndrome type 1 (XLP1), is an inborn error of immunity (IEI) caused by pathogenic variants in *SH2D1A*. The main clinical features of XLP1 include hemophagocytic lymphohistiocytosis (HLH), hypogammaglobulinemia, and lymphoproliferative disorders (LPDs) (1). Approximately 30% of patients with XLP1 present with LPDs, which are often associated with Epstein-Barr virus (EBV) infection (2). 80% of patients with malignant LPD develop B cell non-Hodgkin lymphoma, predominantly affecting the abdomen and cervical regions. The inability of SAP-deficient T cells to recognize antigen-presenting B cells is one of the reasons for the prevalence of EBV-LPD in patients with XLP1 (3). Immune escape due to an imbalance between T helper 1 (Th1) and Th2 responses has also been proposed as a possible mechanism (4). Somatic variants in lymphoma have also been investigated in other IEIs and have shown a distinct genetic signature. In diffuse large B cell lymphomas (DLBCLs) associated with activated PI3Kδ syndrome, common variable immunodeficiency, and DNA repair disorders, somatic variants in genes, including *BRCA2*, *NCOR1*, *KLF2*, *FAS*, *CCND3*, and *BRWD3*, have been reported at a higher frequency compared with non-IEI DLBCLs (5). IEIs are often complicated by tumors that develop at an early age, typically with a median onset around 20 years (6). Cases of LPD without EBV infection in XLP1 have been reported, and the underlying mechanism of their pathogenesis remains unclear. Therefore, this study aimed to elucidate the mechanism of tumorigenesis through transcriptomic analysis of tumor cells and investigation of somatic variants in patients with XLP1-associated LPDs.

## Results

### Patient characteristics

**Figure S1.**
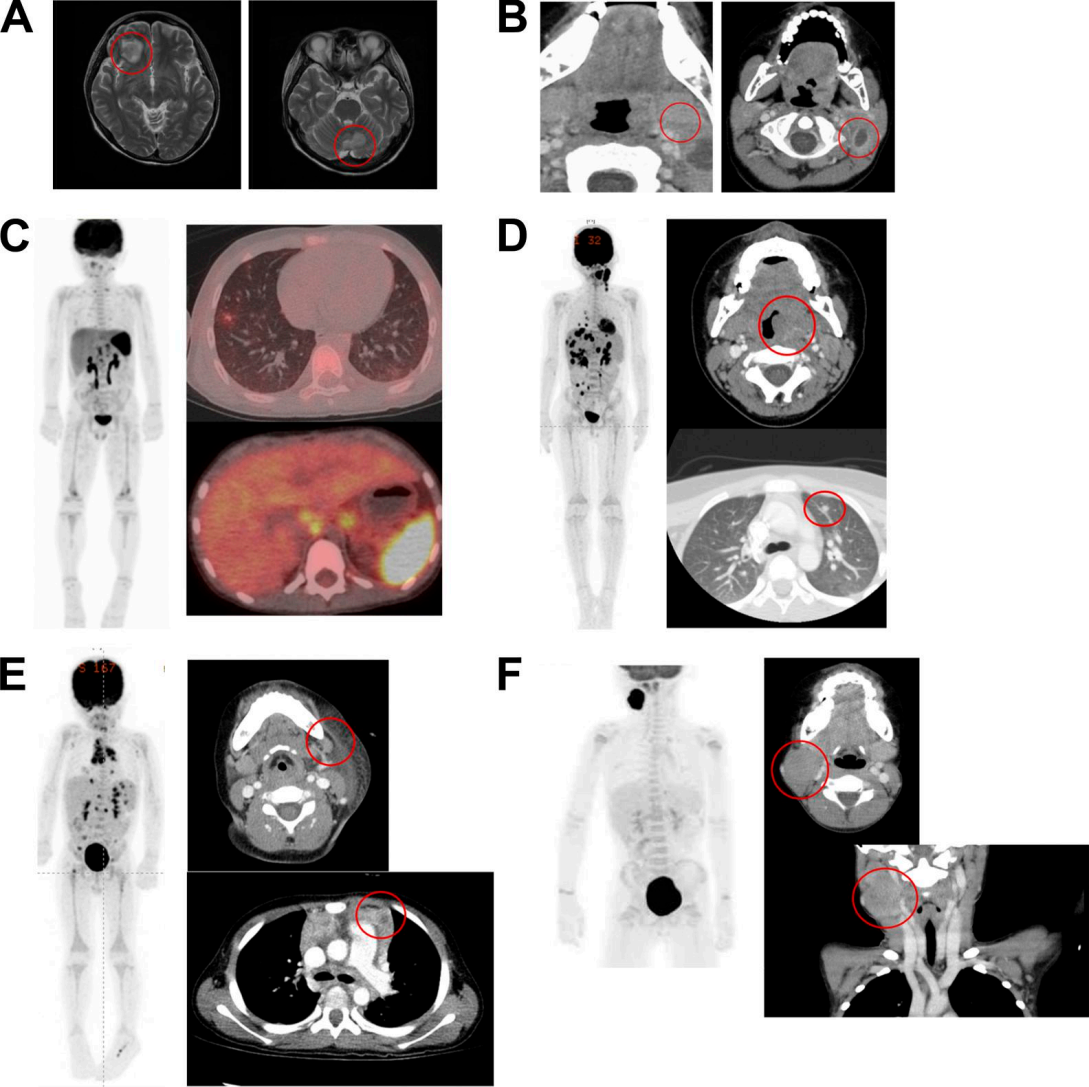
**Imaging findings in patients with XLP1 presenting with LPD. (A–F)** Magnetic resonance imaging T2-weighted images showing abnormal enhancement in the right frontal lobe (left panel) and cerebellum (right panel) in P1 (A). Contrast-enhanced computed tomography (CT) showing multiple enlarged left cervical lymph nodes in P2 (B). An FDG-PET image of the entire body in P3 is shown in the left panel. PET-CT images show high enhancement in the right lung (upper right panel), para-aorta, and liver, with extremely high enhancement in the spleen (lower right panel) (C). An FDG-PET image of the entire body of P4 is shown in the left panel. Contrast-enhanced CT images show enlargement of the left tonsil (upper right panel) and a nodule in the left lung (lower right panel) in P4 (D). An FDG-PET image of the entire body of P5 is shown in the left panel. Contrast-enhanced CT images show enlargement of the left cervical lymph node (upper right panel) and para-aortic lymph node (lower right panel) in P5 (E). An FDG-PET image of the entire body in P6 is shown in the left panel. Contrast-enhanced CT showing enlarged right cervical lymph nodes (upper and lower right panels) (F).

**Figure S2.**
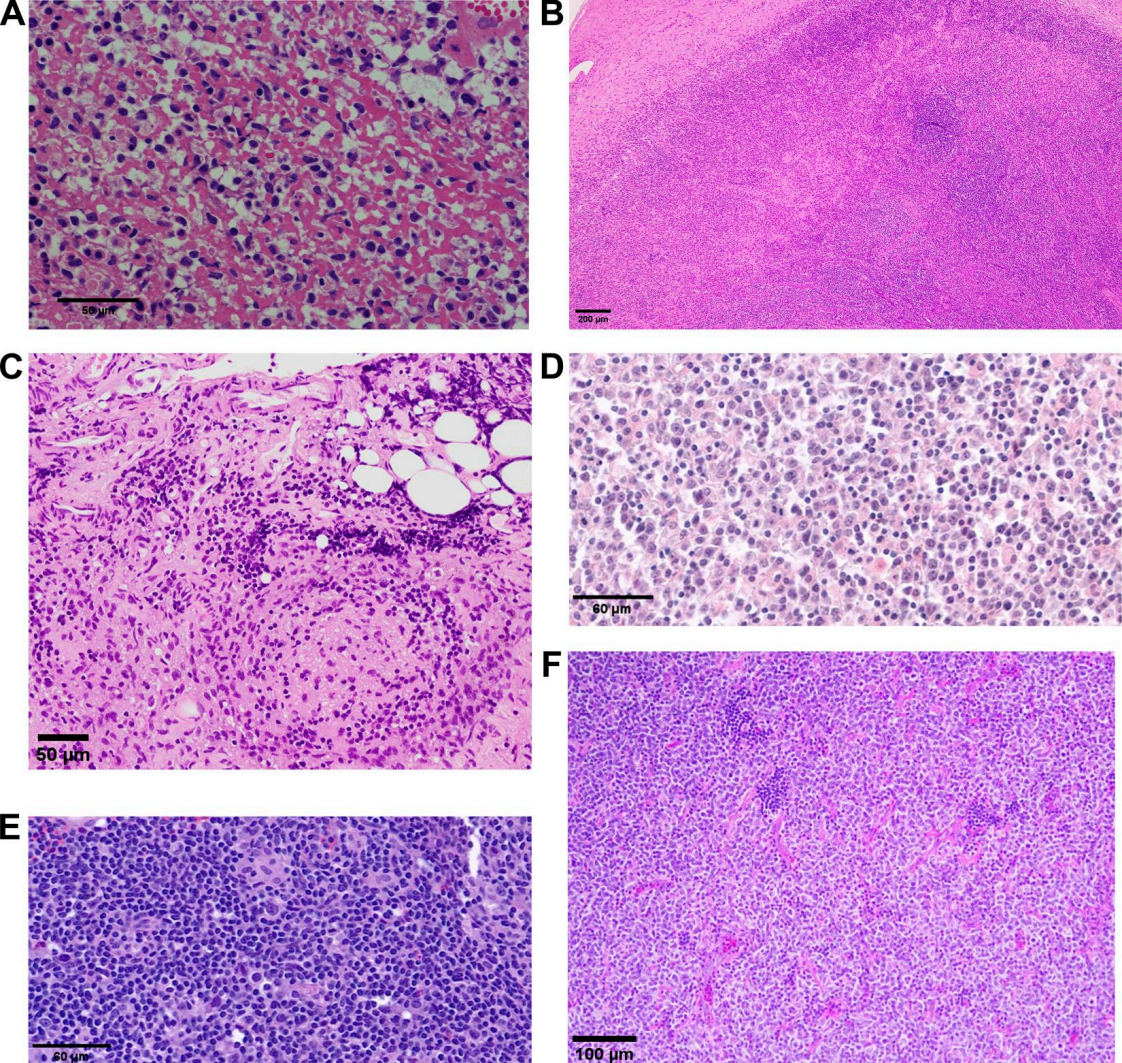
**Hematoxylin and eosin staining of LPD tissues. (A–F)** Panels A to F correspond to the histological images of the LPD tissues from P1 to P6.

**Figure S3.**
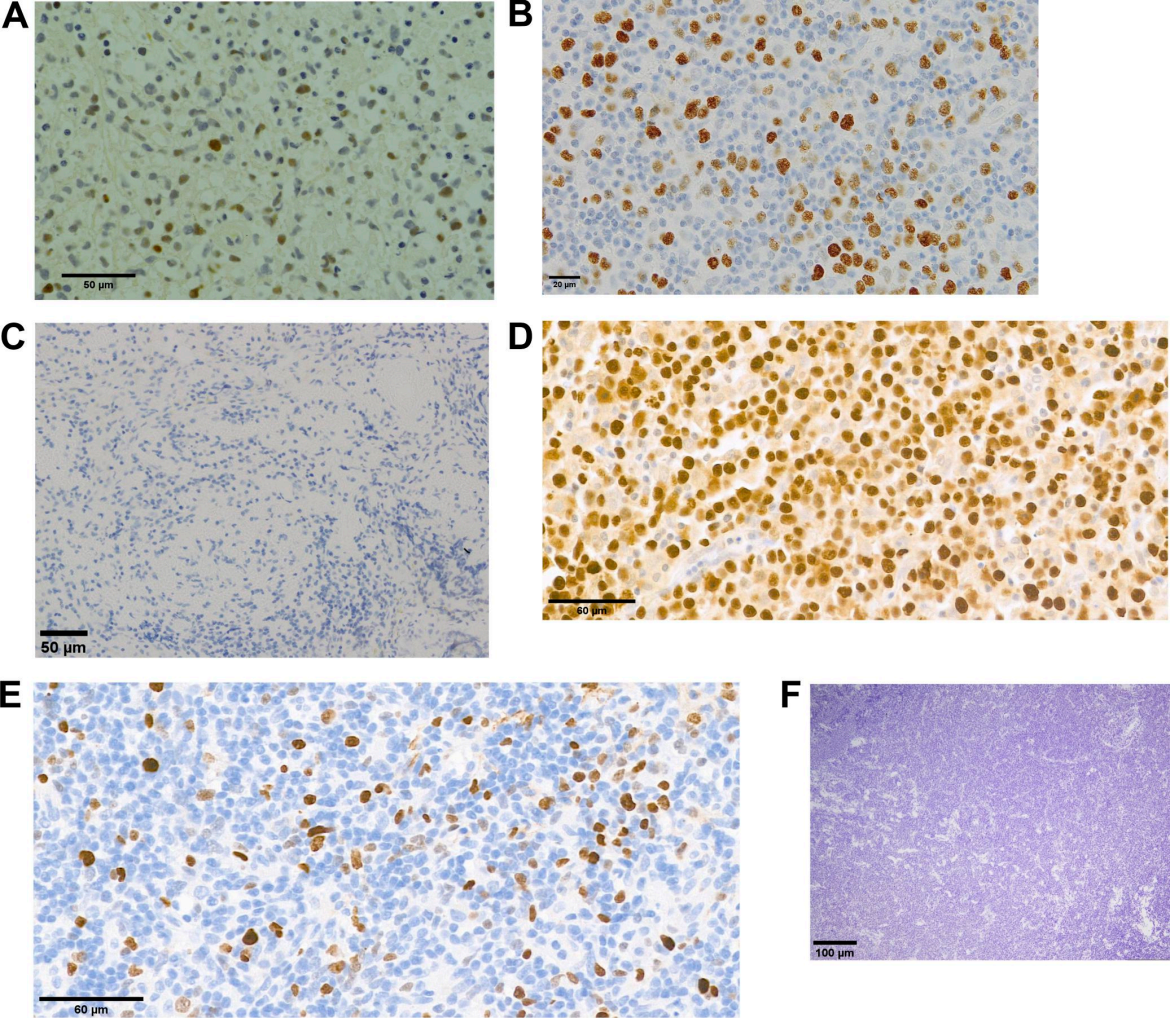
**EBER staining of LPD tissues. (A–F)** Panels A to F correspond to the histological images of the LPD tissues from P1 to P6.

P1 was a male patient with a history of B cell lymphoma at 5 years of age (7). At that time, the lymphoma was considered a primary malignant lymphoma, and since there were no other clinical findings suggestive of XLP, no further investigations for an underlying immunodeficiency were performed. At the age of 18, he developed EBV-HLH, followed by the onset of DLBCL with central nervous system involvement. The patient carried *SH2D1A* c.208_209insC, p.P70fs*4 variant (Table 1). Pathological examination confirmed DLBCL with a cluster of large EBV-encoded small RNA (EBER)–positive B cells (see Fig. S1 A, Fig. S2 A, and Fig. S3 A).

**Table 1.** Patients’ characteristics

Patient	*SH2D1A* variant	XLP symptoms aside from LPD	Age of onset of LPD	Site of involvement in LPD	Surface antigens of infiltrating lymphocytes	EBER positivity in tissue	Pathological diagnosis	Tumor sample type
P1	c.208_209insC p.P70fs*4	HLH	5/18 years	Right frontal lobe and cerebellum	CD20^+^	Positive	DLBCL	Biopsy
P2	Exon 1 deletion	Recurrent otitis media and pneumonia	5 years	Left cervical lymph nodes	CD20^+^, CD10^−^, Bcl6^+^, and MUM1^+^	Positive	LPD (borderline malignancy)	Biopsy
P3	c.162C>A p.Y54X	None	8 years	Liver, intra-abdominal lymph nodes, and subcutaneous nodules	CD3^+^ and CD20^+^	Negative	LPD	Biopsy
P4	Exon 3–4 deletion	Recurrent sinusitis	9 years	Left cervical lymph nodes, lung, liver, and mediastinum lymph nodes	CD20^+^	Positive	DLBCL	FFPE
P5	c.162_201+31delinsTACAAGGACATA	Recurrent pneumonia	5 years	Left cervical lymph nodes and mediastinum lymph nodes	CD30^+^	Positive	Hodgkin lymphoma	FFPE
P6	c.128T>C p.L43P	None	6 years	Right cervical lymph node	CD20^+^, CD10^+^, Bcl6^+^, and MUM1^+^	Negative	DLBCL	Biopsy

P2 experienced recurrent infections from infancy and was diagnosed with hypogammaglobulinemia (8). At 4 years of age, genetic analysis revealed an exon 1 deletion in *SH2D1A* (Table 1). At 5 years of age, the patient presented with left cervical lymphadenopathy. Pathological examination revealed proliferating EBER-positive B cells with preserved follicular structure, suggesting borderline malignancy (see Fig. S1 B, Fig. S2 B, and Fig. S3 B).

**Figure S4.**
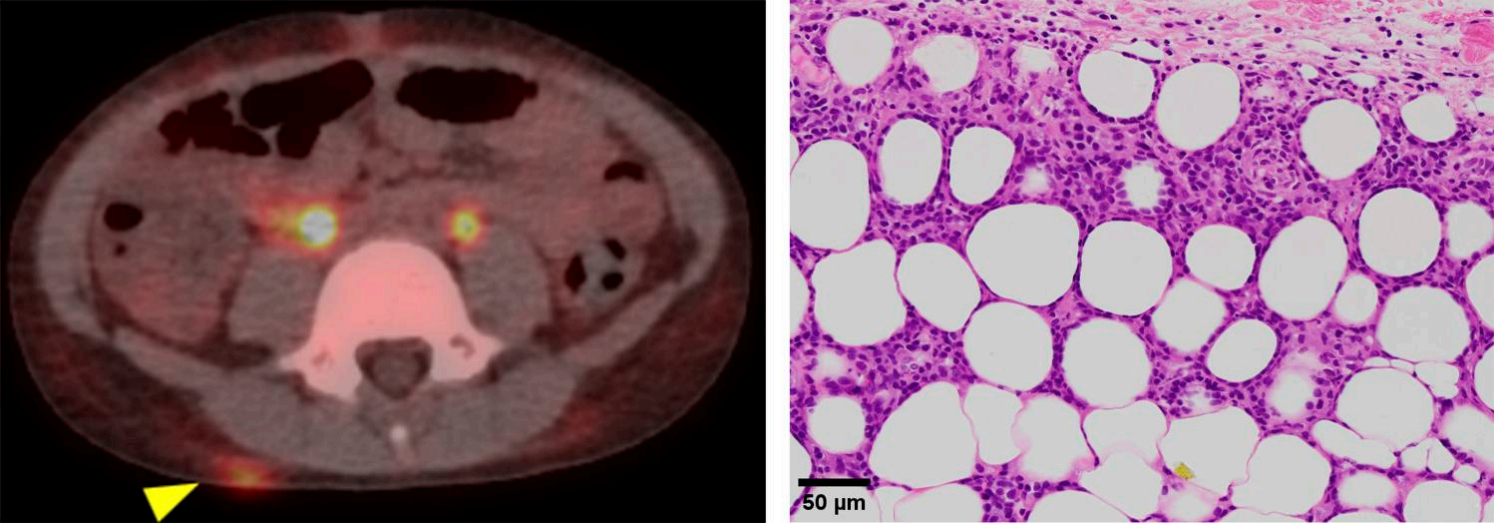
**Imaging findings of subcutaneous panniculitis-like T cell lymphoma in P3.** The FDG-PET scan demonstrated hyperaccumulation in the right lumbar dorsal region (left panel). The right panel shows HE stains of the tumor, with infiltrating lymphocytes identified as CD3^+^ (CD8^+^ > CD4^+^), TIA-1^+^, perforin^+^, ganzyme B^+^, and EBER^−^.

P3 was an 8-year-old boy who presented with fever of unknown origin, lymphatic involvement of the liver, spleen, abdominal cavity, lungs, and subcutis, as well as hypogammaglobulinemia. In the liver, T lymphocytic infiltration was observed around the hepatic and portal veins. Genetic analysis revealed an *SH2D1A* c.162C>A, p.Y54X variant (Table 1). The subcutaneous nodule was diagnosed as T cell lymphoma. An intra-abdominal lymph node biopsy revealed segregation of T cells and EBER-negative B cells, suggesting LPD (see Fig. S1 C, Fig. S2 C, Fig. S3 C, and Fig. S4).

P4 was a 9-year-old boy with a history of recurrent sinusitis who presented with a 2-mo fever, body weight loss, and a sore throat (9). Positron emission tomography (PET) scan revealed increased uptake in the neck, lungs, and intra-abdominal lymph nodes. Biopsy confirmed DLBCL with EBER-positive B cells (see Fig. S1 D, Fig. S2 D, and Fig. S3 D). 1 year later, *SH2D1A* exons 3–4 deletion was identified (Table 1).

P5 had recurrent pneumonia from age 3, and the IgG level was undetectable with normal B cell counts (9, 10). At age 5, the patient had a persistent EBV infection (78,272 IU/ml in the blood sample) and fever, and a PET scan showed increased uptake in the mediastinum and subcarinal lesions. Pathological examination revealed Hodgkin cells and positive for CD30 and EBER staining, leading to a diagnosis of classical Hodgkin’s lymphoma (nodular sclerosis) (see Fig. S1 E, Fig. S2 E, and Fig. S3 F). The patient carried deletion and insertion variants in *SH2D1A* (Table 1).

P6 presented with a right cervical mass at 6 years of age. Biopsy confirmed DLBCL with positive EBER staining (see Fig. S1 F, Fig. S2 F, and Fig. S3 F). Fluorescence in situ hybridization revealed a split signal of *IRF4*. As the brother of the patient also developed lymphoma of colonic origin at 5 years of age, he was tested and found to carry an *SH2D1A* c.128T>C, p.L43P (Table 1). All six patients underwent hematopoietic cell transplantation (HCT) after chemotherapy and have remained in persistent remission.

### Somatic variants in tumor cells

**Figure 1.**
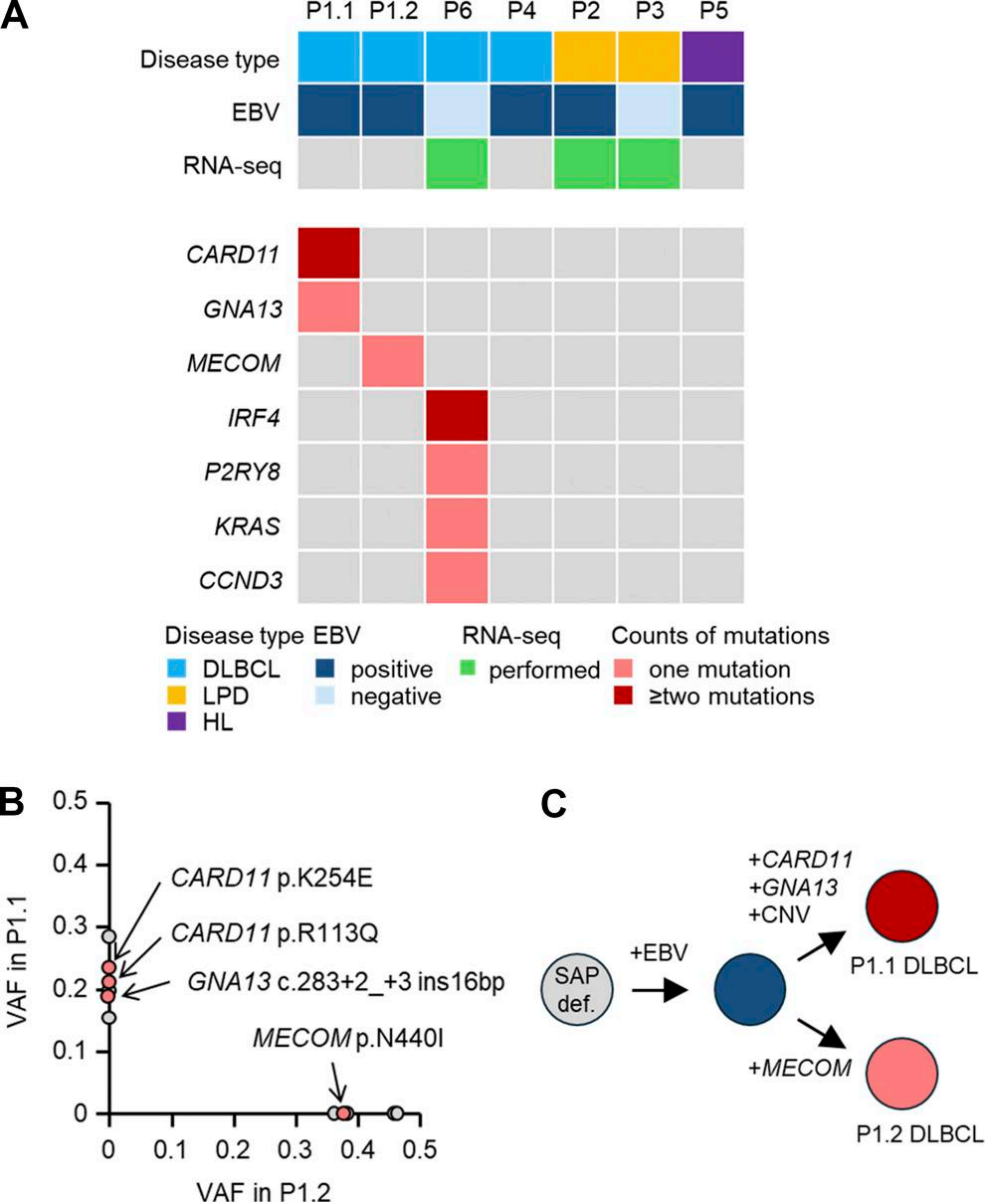
**Patient characteristics and predicted mechanisms of tumor development. (A–C)** Overview of the LPD type of each patient, EBV infection, RNA-seq status, and somatic variants (A). HL, Hodgkin lymphoma. Variant allele frequencies (VAFs) of somatic variants in first and second tumors in P1 are plotted on y and x axes, respectively (B). Distinct clones arose in sequential tumors, driven by different somatic variants plus EBV (C).

A search for somatic variants in LPD samples from P1 to P6 identified 6 non-synonymous variants in the first tumor from P1, 6 in the second tumor from P1, 3 in P4, and 25 in P6 (Table 2). Three variants (*CARD11* [two variants] and *GNA13*) in the first tumor from P1, one variant (*MECOM*) in the second tumor from P1, and 10 variants (*IRF4* [seven variants], *P2RY8*, *KRAS*, and *CCND3*) in P6 were likely pathogenic (Fig. 1). No somatic variants were shared between the first and second tumors in P1.

**Table 2.** All somatic variants in tumor tissues

Patient	Gene	Effect	CDS change	Protein change	CADD phred
P1–1	***CARD11***	Missense	c.G338A	p.R113Q	15.5
	***CARD11***	Missense	c.A760G	p.K254E	21.4
	***GNA13***	Splicing	c.283+2_283+3insCAACGTGATCAAAGG	-	-
	*DENND5A*	Missense	NM_001348749 c.G590A	p.S197N	10.7
	*ADCY2*	Missense	c.G555C	p.E185D	10.1
	*DSP*	Missense	NM_001008844 c.G2968T	p.G990W	18.1
P1–2	***MECOM***	Missense	c.A1319T	NM_001105078 p.N440I	11.4
	*MRC2*	Missense	c.C4315T	p.R1439C	24.4
	*AGPS*	Missense	c.T831G	p.H277Q	13.8
	*CLYBL*	Missense	c.T383C	p.V128A	12.1
	*VBP1*	Missense	c.C62T	p.P21L	13.8
	*SLITRK6*	Missense	c.C1643T	p.S548F	11.5
P4	*BCR*	Missense	c.G153C	p.Q51H	16.1
	*NPIPB2*	Missense	c.C436A	p.L146I	10.7
	*TRPM2*	Missense	NM_001320352 c.C205T	p.P69S	16.8
P6	***IRF4***	Missense	c.A176G	p.K59R	23.1
	***IRF4***	Missense	c.G108T	p.K36N	15.7
	***IRF4***	Missense	c.G177T	p.K59N	20.9
	***IRF4***	Missense	c.G180C	p.Q60H	22.5
	***IRF4***	Missense	c.G181A	p.D61N	36
	***IRF4***	Missense	c.C54A	p.S18R	21.3
	***IRF4***	Missense	c.G38A	p.G13D	28.1
	***P2RY8***	Missense	c.C419T	p.A140V	11.4
	***KRAS***	Missense	c.G38A	p.G13D	27.8
	***CCND3***	Missense	c.A847G	p.T283A	23.1
	*PCLO*	Nonsense	c.C9742T	p.Q3248X	53
	*PAPPA*	Missense	c.G730T	p.A244S	11.6
	*PSG9*	Nonsense	NM_ 001301707 c.C664T	p.R222X	10.5
	*PCNX2*	Missense	c.G5207A	p.R1736Q	37
	*PCID2*	Missense	NM_001127202 c.C1025A	p.A342D	15.5
	*CELSR3*	Missense	c.T5168C	p.L1723P	11.2
	*UHRF1BP1L*	Missense	c.G4258T	p.D1420Y	13.4
	*IGLL5*	Missense	NM_001178126 c.C131T	p.A44V	13.1
	*KIAA0895*	Missense	NM_001199706 c.T365C	p.V122A	10.7
	*COL5A2*	Missense	c.C2426G	p.A809G	18.3
	*DPYD*	Missense	c.T1100G	p.F367C	21.9
	*PCDH15*	Missense	NM_001354420 c.G4565T	p.R1522	13.6
​	*CEP120*	Missense	NM_001166226 c.T1202C	p.L401P	10.3
	*TARBP1*	Missense	c.G1687A	p.E563K	31
	*NALCN*	Missense	NM_001350750 c.C1874T	p.T625I	14.7

P1–1 and P1–2 refer to the first and second tumors from P1, respectively. CDS, coding DNA sequence; CADD, combined annotation–dependent depletion. Genes carrying variants considered pathogenic were shown in bold.

### Chromosome copy number abnormalities in tumor cells

**Figure 2.**
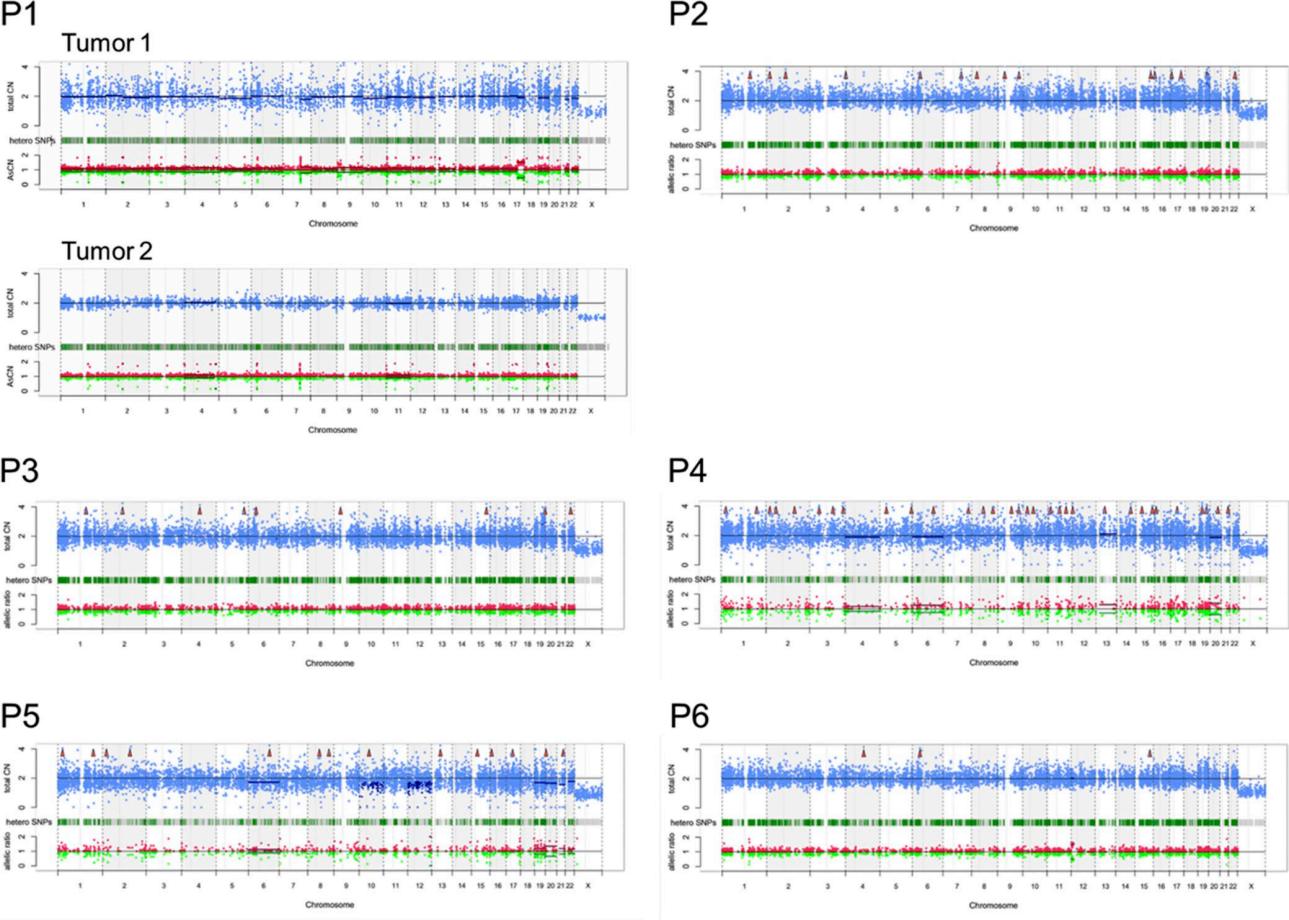
**Chromosome copy number analysis.** Copy number profiles of seven tumors from six patients are shown. Total copy numbers (CNs) are shown in blue, and allele-specific copy numbers (allelic ratio) are shown in red/green.

The first tumor cells from P1 exhibited uniparental disomy (UPD) of chromosome 17q (Fig. 2). No association between 17q UPD and lymphoma has previously been reported. Tumor cells from P6 exhibited UPD of chromosome 12q. This genomic alteration has been observed in follicular lymphoma; however, its role in pathogenesis remains unclear (11).

### Transcriptomic analysis of tumor cells

**Figure 3.**
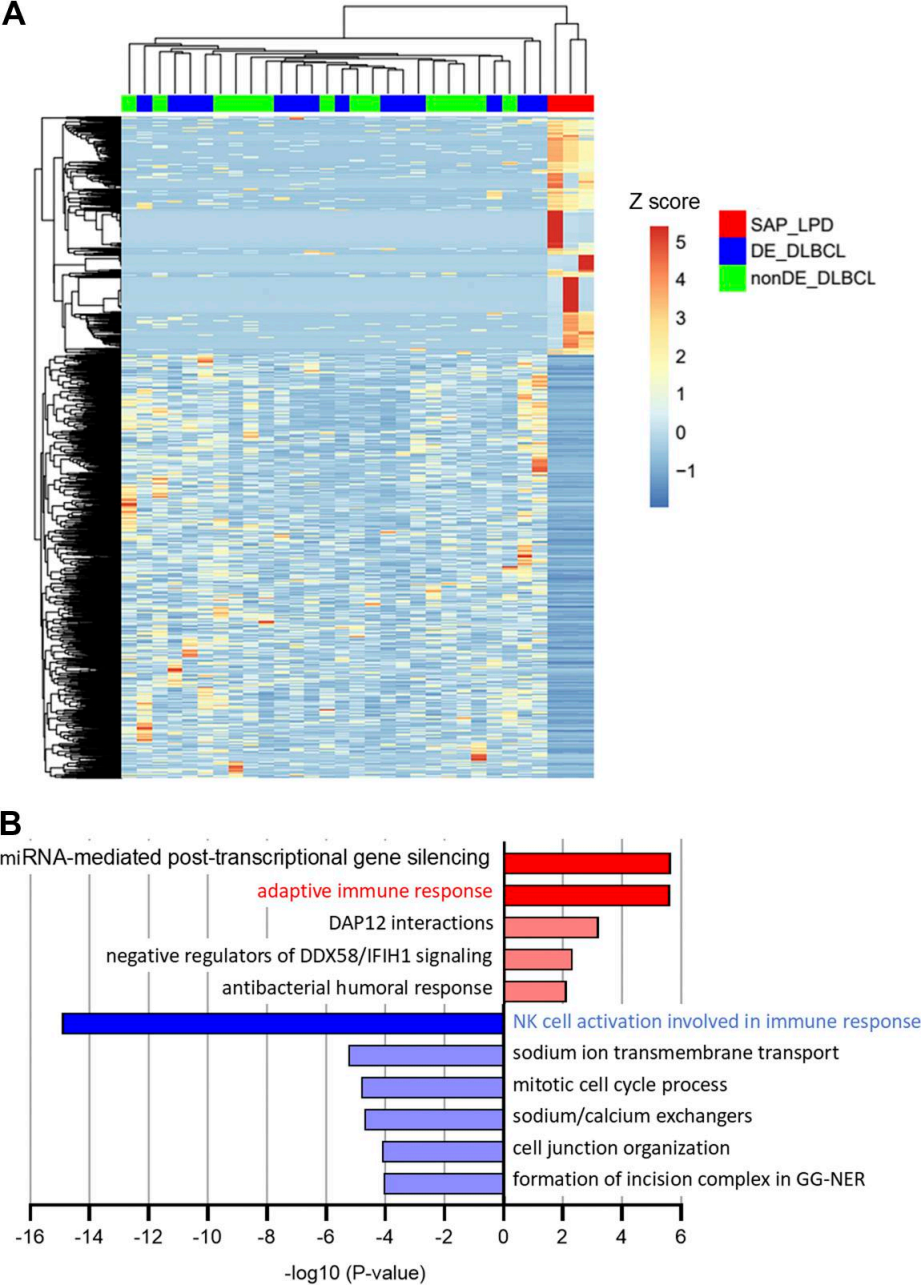
**Comparison of transcriptomes between SAP-LPD and DLBCL. (A and B)** Top 1,548 differentially expressed genes (DEGs) between 2 groups. SAP-LPD and the combined DE-DLBCL and non–DE-DLBCL group were clustered and heat-mapped using Z-scores (A). GO analysis of 552 upregulated (upper) and 996 downregulated genes (lower) was performed using Metascape (9) (B). DE, c-MYC and BCL2 DE.

**Figure 4.**
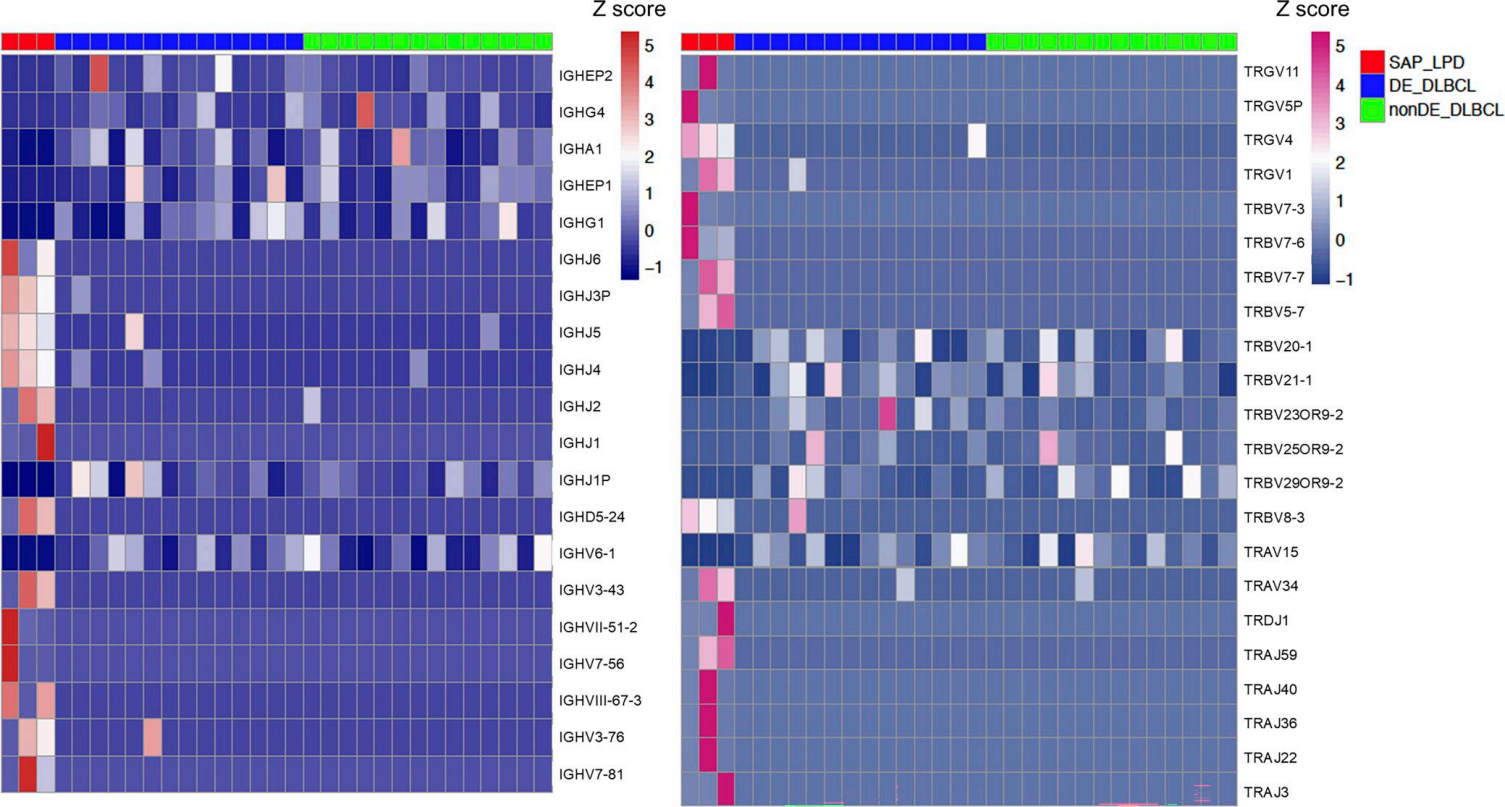
***IgH* and *TCR* genes included in the DEGs.** Genes exhibiting significant differences among the DEGs were listed and heat-mapped using Z-scores. The left panel shows *IgH* genes, and the right panel shows *TCR* genes. DEGs, differentially expressed genes.

**Figure 5.**
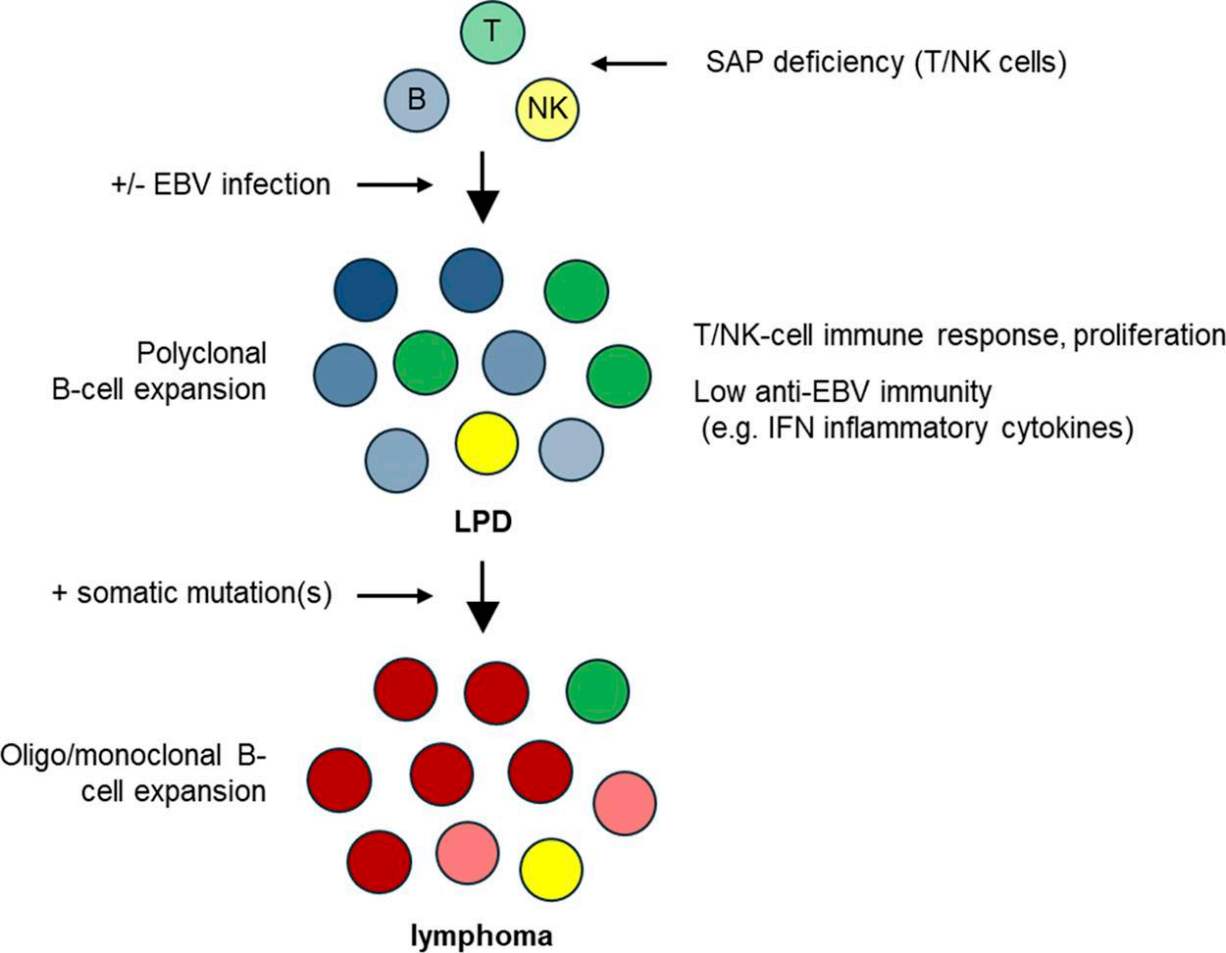
**Proposed mechanisms of lymphoma development in XLP1.** EBV infection, in combination with the intrinsic immunoregulatory defects caused by SAP deficiency, induces polyclonal proliferation of both B and T lymphocytes, resulting in a LPD. The acquisition of somatic variants in this context promotes the emergence and expansion of oligoclonal or monoclonal B cell populations, ultimately leading to the development of lymphoma.

**Figure 6.**
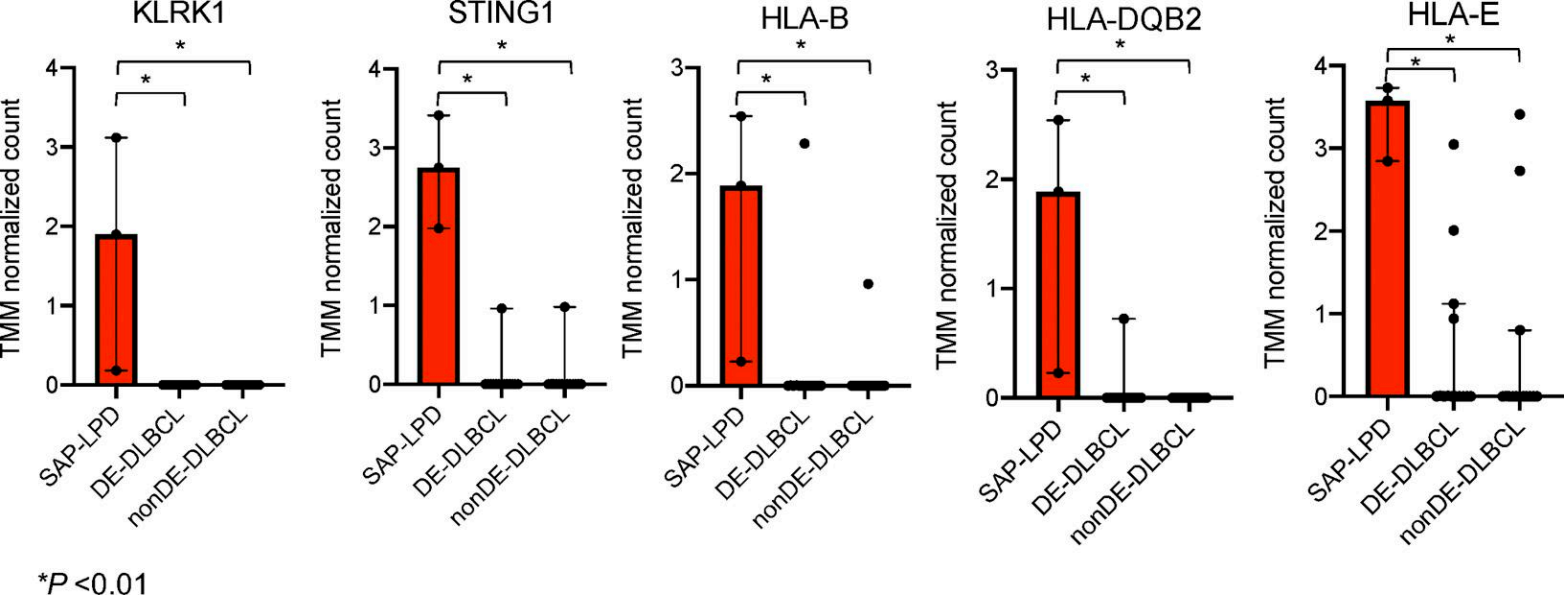
**High expression of *KLRK1*, *STING1*, *HLA-B*, *HLA-DQB2*, and *HLA-E* in SAP-LPD.** Trimmed mean of M values (TMM) normalized counts of *KLRK1*, *STING1*, *HLA-B*, *HLA-DQB2*, and *HLA-E* are shown across three groups: SAP-LPD, DE-DLBCL, and non–DE-DLBCL. Bars represent the median with 95% confidence interval, and individual sample values are plotted as dots. Statistical comparisons between groups were performed using the unpaired *t* test. * indicates a statistically significant difference at P < 0.01.

Tumor RNA samples could not be obtained from P1, P4, and P5. Samples from P2, P3, and P6 were categorized as SAP deficiency-related LPD (SAP-LPD). DLBCL samples from the public data were used as disease controls. An unsupervised clustering analysis was performed between the SAP-LPD and a combined group of c-MYC and BCL2 double expressor (DE)-DLBCL and non–DE-DLBCL (Fig. 3 A). SAP-LPD exhibited a distinct profile compared with DLBCL, suggesting that it forms a separate cluster. Subsequently, Gene Ontology (GO) analysis was performed (Fig. 3 B). In SAP-LPD, pathway analysis revealed a marked downregulation of genes involved in natural killer (NK) cell activation, whereas genes associated with the adaptive immune response were significantly upregulated. Among the differentially expressed genes, various *IgH* and *TCR* genes were upregulated in SAP-LPD, in contrast to a more restricted pattern characterized by upregulation of a single dominant gene in DLBCL (Fig. 4). The increased expression of multiple *IgH* and *TCR* genes in SAP-LPD supports the notion of polyclonal lymphocyte proliferation (Fig. 5). Representative genes from the top 10 GO terms were extracted, and *KLRK1 (NKG2D)*, *STING1*, *HLA-B*, *HLA-DQB2*, and *HLA-E* were identified (Fig. 6).

## Discussion

In P1, the first and second tumors harbored entirely distinct somatic variants, indicating independent origins. Within each tumor, the variant allele frequencies were relatively uniform, supporting clonal homogeneity. Several reports of multiple lymphomas arising from distinct clones support multiclonal lymphomagenesis in XLP1 (12, 13). P2 and P3 exhibited nonmalignant LPDs, consistent with the absence of somatic variants. Despite the malignant nature of the LPDs in P4 and P5, formalin-fixed, paraffin-embedded (FFPE) quality, low tumor purity, or a polyclonal lymphoproliferative background may have limited variant detection. In P5, the low tumor cell content, a characteristic feature of Hodgkin lymphoma, may have influenced the analysis. Alternatively, EBV infection, which was detected in both patients, may have contributed to lymphomagenesis. In contrast, P6 was tumorigenic without EBV infection, and detected somatic pathogenic variants likely drove transformation. Notably, *CCND3*, in which a somatic variant was detected in the P6 tumor, is among the most frequently mutated genes in lymphomas associated with IEIs (5). No recurrent somatic variants unique to XLP1 were detected in this study.

The transcriptome analysis suggests that, unlike conventional DLBCL, which typically arises from monoclonal B cell expansion driven by somatic variants, XLP1-LPD appears to evolve from polyclonal lymphoproliferation. EBV infection under immunosuppression can drive polyclonal B cell proliferation (14, 15). In XLP1, SAP-deficient T cells may fail to recognize EBV-infected B cells, causing reactive polyclonal T cell proliferation (3). Even without EBV infection, intrinsic SAP–T cell defects, such as impaired apoptosis and defective follicular helper T cell function may cause nonspecific B cell activation and expansion (16, 17). In SAP-LPD cases, we observed upregulation of *STING1*, *HLA-B*, and *HLA-DQB2*, suggesting that innate immune activation and enhanced antigen presentation may be common features of the disease. We also observed increased expression of *NKG2D*, an activating receptor expressed on NK cells and cytotoxic T cells that recognize stress-induced ligands on target cells (18). NKG2D ligand activation promotes lymphocyte proliferation and cytotoxicity and is implicated in autoimmunity (19). In SAP-LPD, this pathway may contribute to sustained lymphocyte activation. In contrast, patients with MAGT1 deficiency, who exhibit reduced NKG2D expression, are more susceptible to EBV infection (20). These findings highlight the importance of tight NKG2D regulation in maintaining immune homeostasis and controlling lymphoproliferation.

In contrast, HLA-E, a nonclassical MHC class I molecule, binds to the inhibitory receptor NKG2A on NK cells and suppresses their cytotoxicity (21). EBV-derived LMP1 upregulates HLA-E expression, thereby promoting immune evasion and the development of LPD (22, 23). GO analysis also revealed downregulation of genes involved in NK cell activation. This paradoxical situation of immune activation with impaired EBV response may reflect SAP deficiency. Therapeutic targeting of the NKG2D or NKG2A axis—already been investigated in oncology and autoimmunity—may offer new strategies for XLP1-LPD (18, 24).

**Figure S5.**
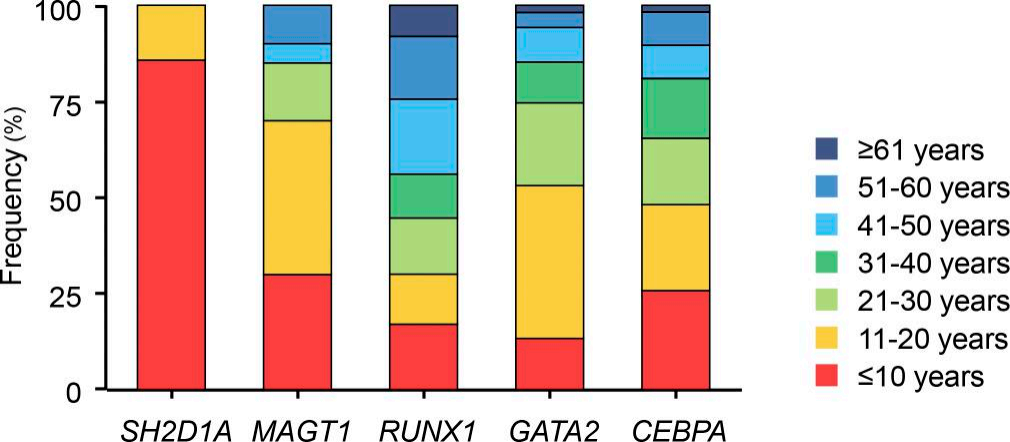
**A comparison of tumor onset age across different monogenic diseases.** Patients with *SH2D1A* variants are from six cases included in this study. Patients with *MAGT1* variants are from (30), and those with *RUNX1*, *GATA2*, and *CEBPA* variants are from (31).

We compared tumor onset age between monogenic immunodeficiency disorders caused by *SH2D1A* and *MAGT1* variants and cancer predisposition syndromes associated with *RUNX1*, *GATA2*, and *CEBPA* variants (Fig. S5). The results highlight the contrast between tumor development driven by impaired T and NK cell function versus tumorigenesis resulting from the intrinsic oncogenic potential of the germline variants. This analysis reveals that tumors develop earlier in immunodeficiencies than in cancer predisposition syndromes. Although HLH remains the most notable prognostic factor for XLP1, lymphoma is also critical (2). Even in non-tumorigenic states, malignant transformation may occur when appropriate triggers are present. Allogenic HCT can be safely performed in asymptomatic patients with XLP1 (25), and early curative HCT is recommended to optimize clinical outcomes.

To our knowledge, this is the first study to identify somatic variants in LPDs in patients with XLP1. However, a major limitation is the small sample size. In addition, in patients with IEIs, it is often difficult to clearly distinguish between lymphoma and LPDs, and in this study, the distinction was made based on histological findings, including preservation of follicular architecture and the presence of monoclonal cell proliferation. Although further validation is required, expression analysis of immune-related molecules such as STING and NKG2D (e.g., by quantitative PCR or immunohistochemistry) in biopsy samples might help clinicians recognize XLP1-LPD. In conclusion, LPD in XLP1 may arise from polyclonal lymphocyte expansion, with tumorigenesis potentially triggered by somatic pathogenic variants. Further studies are warranted to clarify the landscape of XLP1-LPD.

## Materials and methods

### Ethics approval

Genetic analysis was performed after obtaining written informed consent from the patients. This study was performed in accordance with the Helsinki declaration and approved by the Ethics Committee of Institute of Science Tokyo (approval number: G2019-004).

### Whole-exome sequencing

Whole-exome sequencing of paired tumor and control DNA samples was performed. Tumor DNA was extracted from tissue sections or FFPE samples and compared with control DNA extracted from peripheral blood mononuclear cells. Library preparation was performed using the SureSelect Human V6 (Agilent Technologies). Sequencing was performed using an Illumina NovaSeq X Plus system (Illumina) with paired-end 100 bp reads. Somatic mutations were identified using the Gnomon pipeline, with sequencing data of non-paired normal tissues used as controls (26). Copy numbers were detected using the CNACS pipeline (27).

### RNA sequencing (RNA-seq)

Tumor RNA was extracted from the tissue sections. Library preparation was performed using the TruSeq stranded mRNA library (Illumina) following poly A selection. The sequencing was performed on an Illumina NovaSeq X Plus system with paired-end 100 bp reads. Public data on DLBCL (GSE252690) was used. Alignment was performed using Bowtie 2 for RNA-seq data processing (28). Gene expression was quantified using feature Counts. Batch-effect correction was performed using ComBat-Seq, followed by normalization using edgeR. The normalized count data are presented as the trimmed mean of M values normalized counts. Pathway analysis was performed using Metascape (https://metascape.org/gp/index.html#/main/step1) (29).

### Online supplemental material

The supplementary materials include five figures. Fig. S1 shows imaging findings in patients with XLP1 presenting with LPD. Fig. S2 shows hematoxylin and eosin staining of LPD tissues. Fig. S3 shows EBER staining of LPD samples. Fig. S4 shows imaging findings of subcutaneous panniculitis-like T cell lymphoma in P3. Fig. S5 shows a comparison of tumor onset age across different monogenic diseases.

## Consent to participate

Informed consent was obtained from all patients included in this study and their parents.

## Consent for publication

Informed consent was obtained from all participants or their parents.

## Data Availability

The datasets used in this study are not publicly available to protect participant/patient anonymity. Requests to access the datasets can be made to the corresponding author.
